# The effectiveness of problem-based learning compared with lecture-based learning in surgical education: a systematic review and meta-analysis

**DOI:** 10.1186/s12909-023-04531-7

**Published:** 2023-08-01

**Authors:** Qi-Ming Zheng, Yuan-Yuan Li, Qing Yin, Na Zhang, Ye-Peng Wang, Guo-Xiang Li, Zhi-Gang Sun

**Affiliations:** 1 Department of Thoracic Surgery, Jinan Central Hospital, Shandong University, Jinan, 250013 China; 2 Department of Thoracic Surgery, Central Hospital Affiliated to Shandong First Medical University, Jinan, 250013 China; 3 Department of Medical Education, Central Hospital Affiliated to Shandong First Medical University, Jinan, 250013 China; 4 Department of Neurosurgery, Central Hospital Affiliated to Shandong First Medical University, Jinan, 250013 China

**Keywords:** Problem-based learning (PBL), Lecture-based learning (LBL), Surgery, Education

## Abstract

**Background:**

This meta-analysis was conducted to systematically evaluate the impact of problem-based learning (PBL) and lecture-based learning (LBL) teaching models on students’ learning in surgical education.

**Methods:**

We systematically searched the publications related to the application of PBL and LBL in surgical courses in PubMed, Embase, Web of Science and Cochrane Library databases, the last retrieval time is September 20, 2022. After screening the literature according to the inclusion and exclusion criteria, extracting data and evaluating the methodological treatment of the included studies, Stata 17.0 software was used to perform meta-analysis.

**Results:**

Nine studies were included totally. The results showed that compared with LBL, PBL was superior in clinical competence (SMD = 0.81, 95% CI: 0.12 ~ 1.49, P = 0.020) and student satisfaction (SMD = 2.13, 95% CI: 1.11 ~ 3.15, P < 0.0001) with significant differences. But the comprehensive scores (SMD = 0.26, 95% CI: -0.37 ~ 0.89, P = 0.421) and theoretical knowledge (SMD=−0.19, 95% CI: −0.71 ~ 0.33, P = 0.482) to PBL and LBL had no significant difference.

**Conclusion:**

This study showed that the PBL teaching model is more effective than the LBL teaching model in surgical education on the aspects of enhancing clinical competence and student satisfaction. However, further well-designed studies are needed to confirm our findings.

## Introduction

Problem-based learning (PBL), a widely used approach to education and learning, was pioneered in 1969 by Barros, an American professor of neurology at McMaster University in Canada [1]. The first university in the United States to adopted a medical PBL curriculum was the University of New Mexico [2]. Subsequently, some countries from Europe and Asia also begun to promote PBL courses in medical education [3, 4]. In this model, teachers ask questions, take students as the center, mobilize students’ enthusiasm for active learning of knowledge and stimulate students’ innovation and thinking ability. Accordingly, the triggered ability of PBL is to analyze a problem and derive personal learning outcomes, rather than mainly focusing on “solving” the problem, which is the goal of case-based learning (CBL). The theoretical source of the above difference is that PBL relies on constructivism [5] (everyone builds their own knowledge), while CBL relies on cognitivism [6] (the human mind is a problem processor).

Another commonly adopted model is lecture-based learning (LBL), which was firstly implemented in 1894 by the American Medical College Association and American Academy of Medicine [7]. LBL is a traditional didactic pedagogy centered on teachers, with classroom teaching as the main purpose, and knowledge imparting as the goal. It places special emphasis on the importance of theory and knowledge. Compared to the PBL model, in the LBL model, students only receive information from the instructor and attempt to remember the content, rather than understanding concepts and using them [8]. In the 21st century, the choice between PBL and LBL has always been a controversial topic, especially in the medical field.

Among the many medical disciplines, surgery is a highly practical clinical discipline. In clinical practice, surgery is a specialized department with surgical resection and repair as the main means of treatment. Technical ability is the cornerstone of surgery [9]. In addition to having some essential qualities, a component surgeon needs to be technically skilled. Of course, aptitude, interest in surgery, and voluntary motivation also affect learners’ performance in surgical studies [10]. Whether PBL contributes to these traits has been a hotly debated topic. In fact, the current application of PBL in surgical education has not been widely and deeply studied, and the related literature is also relatively little [11]. Moreover, different researchers held different opinions among the effect of PBL compared with LBL in surgical education [12]. Considering the uncertainty of these conclusions, we herein conducted a systematic meta-analysis of the eligible studies to explore the effectiveness of PBL versus LBL in surgical education, aiming to provide guidance for the application and promotion of PBL in surgical education.

## Methods

The meta-analysis was conducted according to the PRISMA guidelines (Preferred reporting items for systematic reviews and meta-analyses) [13]. This protocol has been registered in the International Registry of Prospective Systematic Reviews (PROSPERO) (https://www.crd.york.ac.uk/prospero, CRD42022377288).

### Search strategy

Electronic databases such as PubMed, Embase, Web of Science and Cochrane Library were searched extensively. The last retrieval time is September 20, 2022. Retrieval strategy: The combination of Mesh words (“problem-based learning”, “general surgery”) and corresponding entry terms was used. In addition, in order to obtain all possible relevant studies, the references of the included literature and the relevant literature suggested by each database were manually searched. All articles in the search process have no language restrictions.

### Selection criteria

Inclusion criteria: (1) randomized or quasi-randomized controlled trials; (2) the main research subjects were students involved in surgical studies; (3) the PBL model and the LBL model were used for group teaching respectively, and the teaching effect was compared and studied; (4) the indicators of the outcome are measurement data with sufficient data.

Exclusion criteria: (1) non-original studies (such as meta-analysis, review), expert opinions, meeting summary and repeated studies; (2) no measurement data or insufficient data.

### Data extraction

The data was independently extracted by two reviewers, and any differences can be agreed upon through a consultative discussion with a third researcher. Each included study extracted the following information: (a) first author, (b) publication year, (c) country, (d) study type, (e) age, (f) speciality, (g) number (PBL/LBL), (h) outcome indicators. The objective outcome measurements include comprehensive scores, theoretical knowledge and clinical competence. The subjective outcome measurements include students satisfaction.

### Assessment of study quality

The quality of included studies was assessed by two independent reviewers using Cochrane’s collaborative tool [14], which provides seven criteria to assess the risks of these studies: (a) random sequence generation, (b) allocation concealment, (c) blinding of participants and personnel, (d) blinding of outcome assessment, (e) incomplete outcome data, (f) selective reporting, (g) other biases. According to the description of each study, the assessment of each area is marked as “low risk”, “high risk” or “unclear risk”. Any differences shall be resolved through discussion until consensus is reached. If data is missing, the relevant information can be obtained by contacting the author of the literature.

### Statistical analysis

Meta-analysis was performed using Stata 17.0 software. For measurement data, standardized mean difference (SMD) and its 95% confidence interval (CI) were used as the analysis statistics of the learning effect. The χ² test was used to test the heterogeneity of the results of each study. When P ≥ 0.10 and I²≤50%, the fixed-effects model was used for meta-analysis; otherwise, the random-effects model was used for meta-analysis [15, 16]. Descriptive analysis was used if data could not be combined. Sensitivity analysis was used to determine whether there was heterogeneity, and the results of each study were recalculated using consolidated estimates to see if these recalculations would change the results. Funnel plots, Begg’s and Egger’s tests [17, 18] were used to evaluate publication bias. P < 0.05 is statistically significant.

## Results

### Studies selection and basic characteristics

**Fig. 1:**
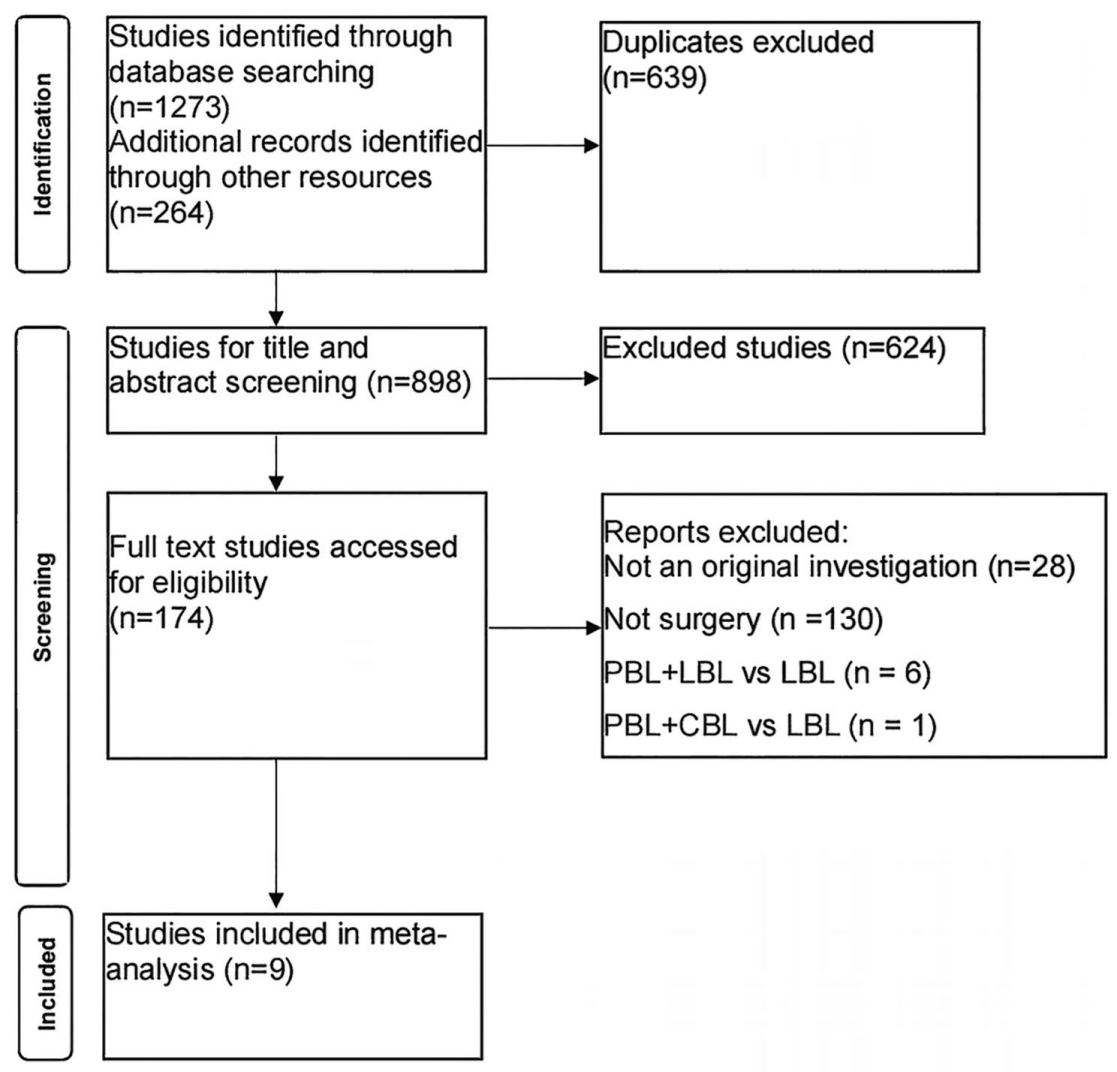
The study selection process

1273 articles were obtained from the initial search, and 264 articles were obtained from the supplementary manual search. After screening according to the inclusion and exclusion criteria, nine literature were finally included for meta-analysis [19–27]. The literature screening process and results are shown in Fig. 1, and the basic characteristics of the included studies are shown in Table 1.

**Table 1:** Basic characteristics of included studies

Author	Year	Country	Study type	Age	Speciality	Number (PBL/LBL)	Outcome
Alleyne	2002	India	RCT	N	Surgery	129 (57/72)	CS
Bai	2017	China	RCT	N	Dental Alveolar Surgery	90 (42/48)	TK, CC, SS
Davari	2021	Iran	RCT	N	General Surgery	175 (72/103)	TK
Holm	1999	Sweden	RCT	N	General surgery, Orthopaedics, Urology	70 (33/37)	CC
Langelotz	2005	Berlin	RCT	25	Surgery	98 (49/49)	CS, CC
Mogre	2014	Ghana	RCT	N	Surgery	175 (82/93)	CS
Qin	2010	China	RCT	N	Oral and Maxillofacial Surgery	231 (118/113)	TK, CC, SS
Tayyeb	2011	Pakistan	Q-RCT	N	Surgery	200 (100/100)	TK, CC
Zhang	2012	China	RCT	N	Oral and Maxillofacial Surgery	87 (43/44)	TK, CC, SS

Abbreviation: RCT, randomized controlled trial; Q-RCT, quasi-randomized control trial; PBL, problem-based learning; LBL, lecture-based learning; CS, comprehensive scores; TK, theoretical knowledge; CC, clinical competence; SS, student satisfaction

### Evaluation of methodological quality included in the study

**Fig. 2:**
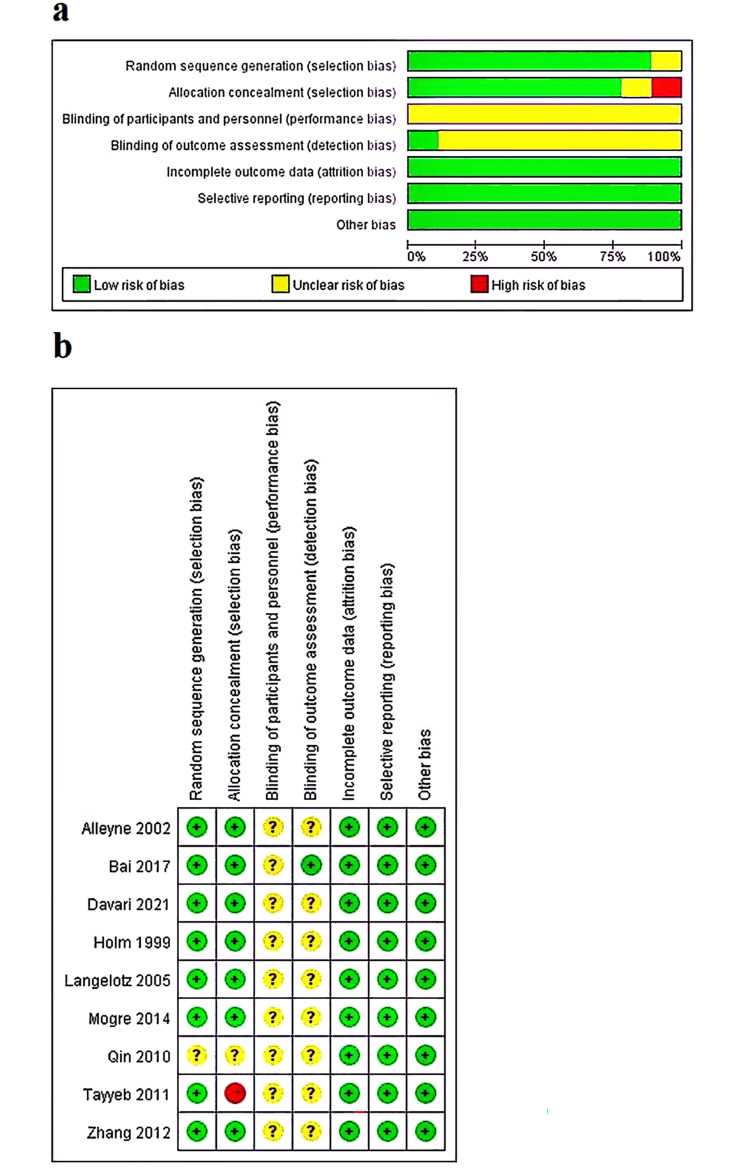
Risk of bias assessment. (**a**) Risk of bias graph as percentages for all included studies; (**b**) Risk of bias summary for each included study

The assessment of bias in nine articles is shown in Fig. 2. The author shows the results of each quality project as a percentage of cross study. In one study [26], the allocation sequence of its was generated by the preference of the students, whom were assigned to the experimental group or control group; this study was therefore judged to be high risk in this domain. All articles reported complete outcome data and no selective reporting. According to the definition of the Cochrane Cooperation Organization, all studies seem to have no “other sources of bias”. In general, most of the included articles were found to have low bias risk and high quality risk (Fig. 2).

### Meta-analysis result for comprehensive scores

**Fig. 3:**
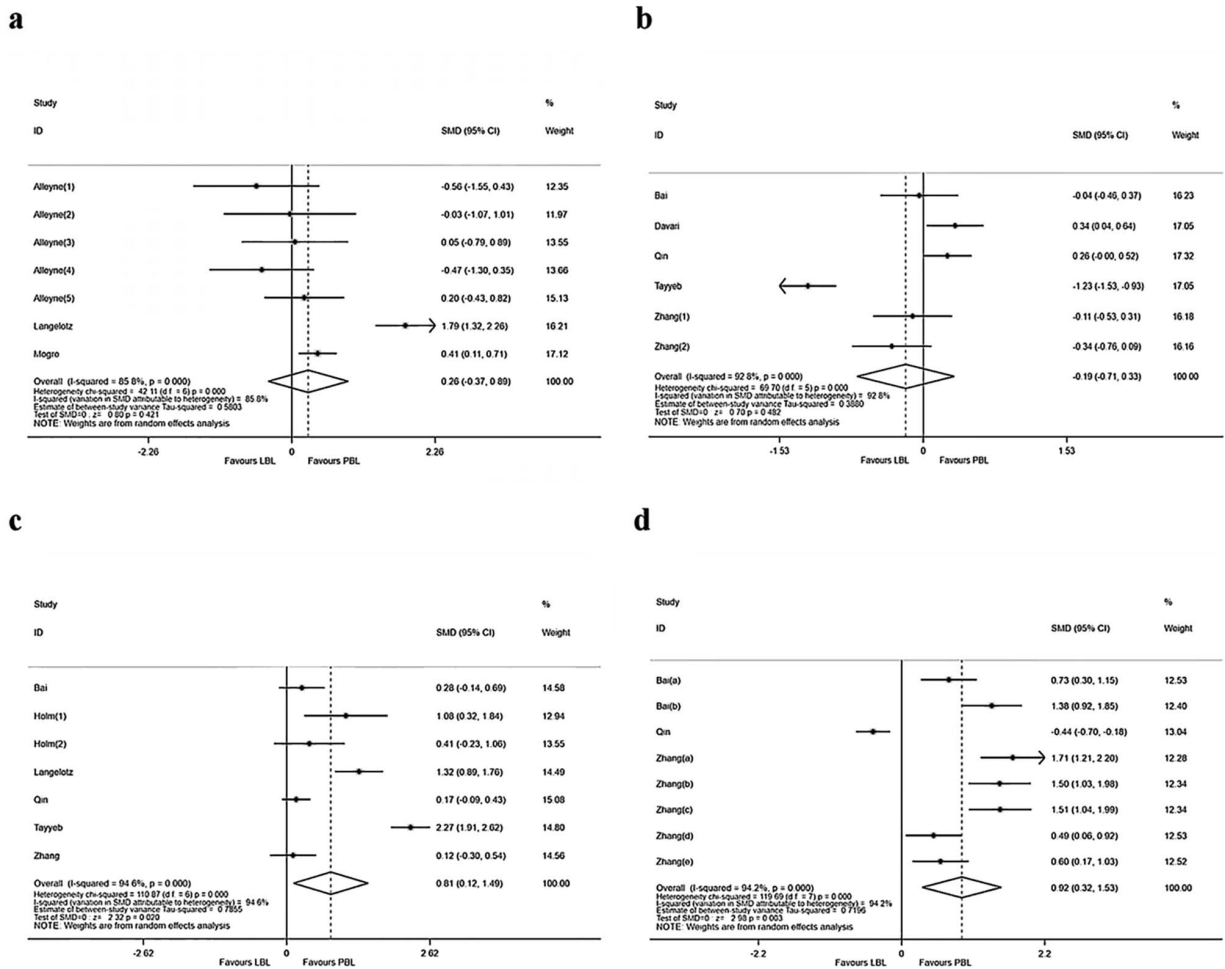
Meta-analysis of the effectiveness of problem-based learning compared with lecture-based learning in surgical education: (**a**) comprehensive scores, (**b**) theoretical knowledge, (**c**) clinical competence, (**d**) student satisfaction

Three articles (7 studies) investigated the comprehensive scores of 332 patients [19, 23, 24], including 118 cases in the PBL group and 214 cases in the LBL group. There was statistical heterogeneity among the results (P<0.001, I²=85.8%), so the random effect model was used for meta-analysis. The results showed that there was no significant difference in comprehensive scores between students in PBL group and LBL group (SMD = 0.26, 95% CI: -0.37 ~ 0.89, P = 0.421) (Fig. 3a).

### Meta-analysis result for theoretical knowledge

Five articles (6 studies) investigated the theoretical knowledge of 870 patients [20, 21, 25–27], including 418 cases in the PBL group and 452 cases in the LBL group. There was statistical heterogeneity among the results (P = 0.000, I²=92.8%), so the random effect model was used for meta-analysis. The results showed that there was no significant difference in surgical theoretical knowledge between students in PBL group and LBL group (SMD=-0.19, 95% CI: −0.71 ~ 0.33, P = 0.482) (Fig. 3b).

### Meta-analysis result for clinical competence

Six articles (7 studies) investigated the clinical competence of 776 cases in total [20, 22, 23, 25–27], including 385 cases in the PBL group and 391 cases in the LBL group. This objective outcome measurement includes operation skill, case analysis and other clinical projects. Because the survey items of the six articles are different and have significant heterogeneity (P<0.001, I²=94.6%), only the random effect model is used for combined analysis of clinical competence. The results showed that the clinical competence of medical students in PBL group was better than that in LBL group (SMD = 0.81, 95% CI: 0.12 ~ 1.49, P = 0.020) (Fig. 3c).

### Meta-analysis result for student satisfaction

Three articles (8 studies) investigated the student satisfaction of 846 cases in total [20, 25, 27], including 417 cases in the PBL group and 429 cases in the LBL group. This subjective outcome measurement includes learning interest, collaboration motivations and other projects. Given the differences in the survey items of the three articles and the significant heterogeneity (P<0.001, I²=94.2%), the random effect model is used for combined analysis of overall satisfaction. The results showed that the student satisfaction in the PBL group was better than that in the LBL group (SMD = 0.92, 95% CI: 0.32 ~ 1.53, P = 0.003) (Fig. 3d).

### Sensitivity analysis

**Fig. 4:**
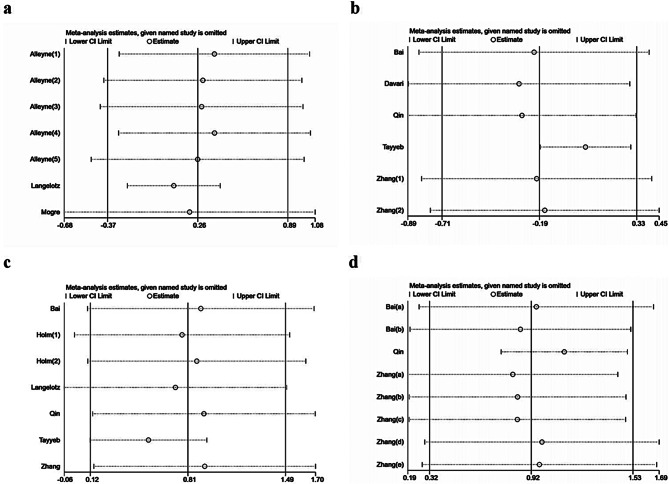
Sensitivity analysis of the effectiveness of problem-based learning compared with lecture-based learning in surgical education: (**a**) comprehensive scores, (**b**) theoretical knowledge, (**c**) clinical competence, (**d**) student satisfaction

Because of high heterogeneity, sensitivity analysis was implemented to evaluate the reliability of the results (Fig. 4). After excluding the study with the largest weight [24], the pooled effect size was in favor of the intervention group (SMD = 0.20, 95% CI: -0.68 ~ 1.08, P = 0.652) for comprehensive scores and did not change the effects observed in the primary analysis. In addition, after excluding the study with the largest weight [20], the pooled effect size in theoretical knowledge, clinical competence and student satisfaction was in favor of the intervention group (SMD=-0.28, 95% CI: -0.89 ~ 0.33, P = 0.369; SMD = 0.92, 95% CI: 0.14 ~ 1.70, P = 0.021; SMD = 1.12, 95% CI: 0.74 ~ 1.50, P < 0.00001), which also did not change the effects observed in the primary analysis. The results of sensitivity analysis indicated the relative stability of our results.

### Publication bias

**Fig. 5:**
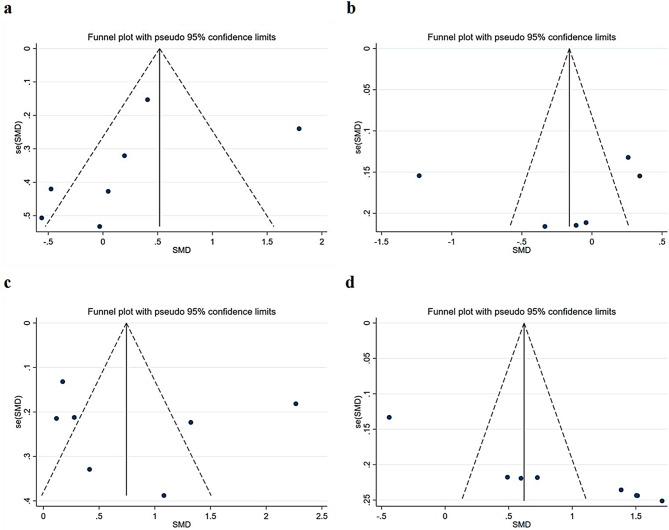
Funnel plot of the effectiveness of problem-based learning compared with lecture-based learning in surgical education: (**a**) comprehensive scores, (**b**) theoretical knowledge, (**c**) clinical competence, (**d**) student satisfaction

The evaluation of publication bias was conducted using a funnel plot for each pooled outcome indicator (Fig. 5). The shape of the funnel plot did not show asymmetry, preliminarily indicating the absence of any publication bias. Considering that the number of articles included in the meta analysis is too small to judge the symmetry of the funnel plot [28]. In addition, the subjectivity of visual evaluation itself cannot be ignored. We further used the Begg’s rank correlation test and Egger’s linear regression test to evaluate the publication bias of comprehensive scores (Z = 0.90, P = 0.368; t=-0.99, P = 0.368), theoretical knowledge (Z = 1.13, P = 0.260; t=-0.27, P = 0.797), clinical competence (Z = 0.60, P = 0.548; t = 0.36, P = 0.737) and student satisfaction (Z = 3.09, P = 0.002; t = 8.40, P<0.001). When studying student satisfaction, there was publication bias in the pooled results. Then we used the shear patching method and did not find that the elimination of individual studies changed the original merging results. The above discussion showed that the results were relatively true.

## Discussion

In recent years, due to continuous exploration by researchers, a growing body of research has highlighted the effectiveness of PBL in medical education. Based on such research, a large, systematic, and comprehensive meta-analysis of the PBL teaching mode was conducted in the context of standardized residency training in China, which suggested that PBL positively affects the mastery of theoretical knowledge, clinical diagnostic thinking, teamwork skills, analytical and problem-solving skills, consulting documents, learning interests and learning ability; however, it does not offer these advantages with respect to improving self-directed learning ability, communication ability or practical ability. This meta-analysis also provided ideas for further research on teaching methods that are suitable for different majors and abilities [29]. Based on this single-country study, we broadened the search and specifically explored the effectiveness of PBL and LBL in surgical education with the aim of providing surgical faculty and residents with new avenues for pedagogical reform.

This research shows that the use of PBL, as opposed to LBL, in surgery education can not only enhance students’ clinical competence but also effectively improve their satisfaction with learning. However, no significant differences are observed between the two teaching models with respect to comprehensive scores and theoretical knowledge. In contrast to the traditional teaching model, a core idea of PBL is that learning situations activate prior knowledge and promote the learning of new knowledge. This situation is similar to the demand for knowledge in the real world. In this way, students can increase their likelihood of recalling and applying information stored in their memory [30]. Moreover, the PBL teaching model pays more attention to the cultivation of students’ autonomous learning ability, theoretically eliminates the drawbacks of LBL model such as teaching rigidity and single means, fully draws out students’ ability to analyse problems, and improves teaching and learning quality. This model is also believed to effectively improve students’ professional knowledge and enrich their team cooperation experience [31]. One study showed that the implementation of PBL in medical schools not only generates implicit clinical knowledge and judgements but also provides the confidence and preparation necessary to convey independence [32].

In this study, students in the PBL group were not superior to those in the LBL group with regard to acquiring theoretical knowledge, which was consistent with the research results reported by Schwartz et al. [33] at Kentucky University. Some previous studies have also shown that PBL students do not perform well in knowledge tests, and so the traditional teaching model has been recommended to ensure content coverage [34–36]. The PBL teaching model views the problem as the centre of learning and the learner as the main relevant body. In the process of solving problems, students focus on the problem to locate and acquire relevant basic theoretical knowledge and ultimately to enhance their personal learning outcomes by analysing the problem. This model can effectively stimulate the learning interest of scholars and fully mobilize and take advantage of learning initiative. A disadvantage of this model is that learners must spend more time and energy in the process of acquiring and mastering knowledge and are more likely to miss key knowledge. According to the LBL teaching model, teaching is conducted based on a vertical knowledge system, which is relatively comprehensive and systematic. In a clinical course, with regard to the depth and breadth of each single course as well as the knowledge structure of the subjection stage, the PBL model can promote interaction between knowledge acquisition and practical ability improvement; however, compared with the LBL model, the gap between the two in acquiring theoretical knowledge is not obvious. However, when we interpret these results, we should note that many factors affect test scores pertaining to theoretical knowledge. Feeley et al. claimed that motivation, learning skills, learning methods and other important factors must be considered [37], which makes it difficult to draw reliable conclusions concerning the effects of PBL and LBL.

In this study, the surgical clinical competence of students in the PBL group exhibited significant improvement compared with that of students in the LBL group, which was consistent with Walker’s research results [38] and contrary to the research conclusions drawn by Albanese et al. [39]. The PBL teaching model can encourage students to actively learn knowledge and organically transform theoretical knowledge into practical ability. Under the guidance of teachers, the PBL teaching model shapes the knowledge summarized by the group by defining learning objectives, proposing surgical problems, establishing case assumptions, collecting data, and discussing and analysing specific cases with the goal of ensuring that students can understand the acquired knowledge more thoroughly and remember it more firmly, improve their ability to solve the practical problems they encounter in surgical clinical practice, acquire effective surgical clinical reasoning experience, and cultivate good thinking habits. Combining the knowledge learned in the classroom with the clinical practice skills learned in the hospital, properly applying this method to the surgical clinical practice teaching process can greatly improve students’ theoretical knowledge and clinical practice skills in the context of surgery and lay a solid foundation for training students to become qualified clinicians in the future. Similarly, we found that student satisfaction in the PBL group exhibited significant improvements compared with student satisfaction in the LBL group, which was consistent with the conclusions of some previous research. In this context, McGregor et al. showed that the PBL course can stimulate the enthusiasm of students and teachers. Unfortunately, this stimulation does not translate into more effective knowledge dissemination [40]. Centres using the PBL method have found that this approach can improve students’ enthusiasm and fun, but no convincing evidence has been found to indicate that it improves their learning [12].

To date, PBL has been proven to have advantages over LBL in various fields of medicine, such as gynaecology and obstetrics [12], internal medicine [41], anatomy [42], pathology [43], and medical cell biology [44]. During the COVID-19 pandemic, the forms of teaching used in PBL courses have also undergone tremendous changes. Researchers have found that students who take online PBL courses exhibit lower performance than those who participate in the traditional face-to-face method of teaching [45]. However, in Nigeria, researchers have shown that computers and up-to-date libraries as well as internet and audio-visual facilities can enhance the adaptation of PBL to medical courses [46]. Nevertheless, one study found that both in-person PBL and virtual PBL were preferable to lectures with regard to preparing students for NBME examinations and surgical cases [47]. Many more reliable studies are needed to prove the effectiveness of online PBL courses. Due to the continuous deepening, development and reform of the educational model, researchers and teachers have found that the combination of some other teaching models with PBL in surgical education also have good teaching effects. Relevant studies have noted that combining PBL with CBL can improve the performance of medical students and residents and clinical skills in thyroid surgery [48]. The integration of PBL and LBL led by residents is preferable to lectures led by teachers, and this approach can prepare students for examinations and probation experience; it may thus serve as a useful aid for clinical education [47]. Accordingly, we believe that the PBL and LBL dual track teaching model can be used for surgical education under conditions of limited medical colleges and teachers. For one thing, LBL is used to help students master theory and train comprehensive practical skills in the context of surgical theory and basic surgical skills. In addition, PBL is used for surgical operation and case analysis, which can improve the clinical competence and capabilities of students. Combining the knowledge learned in the classroom with the clinical practice skills learned in the hospital and properly applying this method in the surgical clinical practice teaching process can greatly improve students’ theoretical knowledge and clinical practice skills in the context of surgery and lay a solid foundation for training students to become qualified clinicians in the future.

It must be noted that our study has the following limitations: (1) the quality of some of the included literature is not high, which may impact the results of the analysis; (2) the randomization method and allocation for inclusion in the study are not clear, so selective bias may be an issue; (3) the degree of difficulty of the specialized courses and test questions included in the study varies, which may affect the accuracy of the results; (4) the publication bias found by Egger’s test may affect the authenticity of some pooled results; and (5) the heterogeneity is significant. According to the information provided in the literature, no clear reason for heterogeneity has been found, and many factors may lead to heterogeneity. First, the methods used to implement PBL in medical colleges and universities are not uniform, such as the time distribution of each PBL program. Second, the organizer who actually teaches students represents another potential contributor to the heterogeneity because the learning process may be seriously affected by teachers’ performance. Third, the learning habits of students are also an important source that is difficult to unify. Despite these limitations, this meta-analysis is helpful with regard to our ability to understand the effectiveness of problem-based learning versus that of lecture-based learning in surgical education.

## Conclusion

PBL has emerged as a prevalent educational model in various medical schools across different countries, gradually showcasing its strengths in some aspects. In this study, we tentatively explored the effectiveness of PBL compared with LBL in surgical education. Based on the results of our research, the available evidence supports PBL as more effective than LBL on the aspects of enhancing clinical competence and student satisfaction. However, in terms of comprehensive scores and theoretical knowledge, our results showed PBL does not have a significant advantage over LBL. Therefore, we believe that it is necessary to adopt the PBL teaching model in surgical education. In future research work, high-quality studies with larger sample sizes and standardized designs are needed to further verify this finding. Notably, with the continuous deepening and development of teaching reform in medical education, the feasibility and necessity of popularizing joint teaching models such as the combination of PBL and LBL methods also deserve further exploration.

## Data Availability

All data generated or analyzed during this study are included in this published article. The Stata raw dataset can be provided on request. The corresponding author, Zhi-Gang Sun, will provide additional data, if requested.

## Abbreviations

PBL: Problem-based learning
LBL: Lecture-based learning
PRISMA: Preferred Reporting Items for Systematic Reviews and Meta-Analysis Protocols
SMD: Standardized mean difference
CI: Confidence interval
CBL: Case-based learning
